# Understanding *Love* in the L1 and the Additional Language: Evidence from Semantic Fluency and Graph Analysis

**DOI:** 10.3390/jintelligence14010003

**Published:** 2025-12-24

**Authors:** Maria Pilar Agustín Llach

**Affiliations:** Department of Modern Philologies, Universidad de La Rioja, 26004 Logroño, Spain; maria-del-pilar.agustin@unirioja.es

**Keywords:** emotion love, EFL, monolingual and bilingual learners, network analysis

## Abstract

This study explores how adolescent learners conceptualize the emotion of love in their first language (Spanish) and in English as a foreign language (EFL), comparing monolingual Spanish speakers and Spanish–Arabic bilinguals. A total of 66 participants (33 per group), all with A2 proficiency in English, completed a semantic fluency task in both Spanish and English, producing as many words as possible in relation to the prompts Amor and Love. The data were analyzed using graph theory to capture the organization of participants’ emotion lexicons. The results show that love is a highly productive and cohesive semantic field, eliciting significantly more responses in L1 than in L2, for both Spanish-only (t = −8.866, *p* < 0.001) and Spanish–Arabic (W = 101.0, *p* = 0.001) participants. The differences between the two learner cohorts were not significant in Spanish nor in English. The results from the graph analyses revealed that learners displayed rich and strongly connected networks in Spanish, with learners with a migration origin showing slightly more fragmented networks. In English, both groups performed similarly, with responses probably mediated by L1 translation equivalents and metaphorical associations (e.g., *heart*, *flower*, and *red*). The findings suggest that emotional lexicons are better developed and more efficiently organized in the L1, whereas FL representations are shaped by proficiency, context of learning, and reliance on L1 conceptual structures. This study contributes novel insights into bilingual and heritage learners’ emotional conceptualization and highlights the value of graph analysis for examining the structure of emotion words.

## 1. Introduction

Semantic categorization is an activity which humans engage in in order to make sense of the world. Semantic categorization consists in grouping elements, words, concepts, and emotions, based on, mainly, their semantic similarities. In broad terms, we can distinguish among taxonomic, ahoc, schema-like, experiential, and emotional categories. Taxonomic categories are based on hierarchical relationships of memberships and shared semantic features. In other words, the members of the category establish an organization based on a hierarchy of membership going from superordinates to base words and subordinates. Taxonomic categories reflect biological or natural groups; thus, some examples of these categories typically include animals (cat, dog, and lion), fruits and vegetables (apple, banana, tomato, and potato), or flowers (rose, daisy, and lily) (cf. Hernández Muñoz et al., 2025; Mirman et al., 2017). Other categories, based on thematic relations of contiguity or co-occurrence in events or scenarios, can be called schematic categories, such as countryside. Other semantic categories based on different types of experiences include ad hoc categories, made up specifically for the task, such as yellow things, things on the table (cf. Hernández Muñoz et al., 2025), or emotional categories, where participants are asked to react to stimuli such as love, hate, or COVID (cf. Blanco, 2022).

Emotion plays a significant role in language, influencing both how we express ourselves and how we understand language. From a linguistic perspective, emotions are expressed through various means, including vocabulary choices, grammatical structures, and prosodic features like intonation. Furthermore, emotions impact language processing, affecting comprehension and memory (cf. Ferré et al., 2010), and even shaping how we conceptualize and categorize emotions (Blanco, 2022). Emotion also plays a relevant role in foreign language learning, with positive emotion found to enhance and improve acquisition (Frances et al., 2020).

## 2. Expressing and Understanding Emotion in L2 vs. L1

Our understanding of emotions is shaped by language. For example, the way we categorize and label emotions in our language can influence how we experience and perceive them.

Research distinguishes among emotion-label words, which denote emotions such as *sad* or *happy*, and emotion-laden words, which, while not explicitly naming an emotion, can evoke feelings or reactions, such as a *cake*, *kiss*, *candy*, *gun*, or *cancer* (Sabater et al., 2023; Grünewald Soto & Osorio, 2010). Accordingly, research (cf. e.g., Yao et al., 2023) has identified five main basic or biological emotions, which are universal: fear, anger, happiness, sadness, and surprise. Additionally, complex emotions are blends of basic emotions or more nuanced feelings that involve cognitive processing and vary across individuals and cultures such as love, grief, regret, shame, jealousy, and guilt (e.g., APA Dictionary of Psychology, n.d.; Bisquerra Alzina et al., 2015; Blanco, 2022). These may be influenced by personal experiences and cultural factors and often require cognitive processing.

It is a general belief that emotions have a universal component, but also a sociocultural and linguistic one, so that the specific sociocultural and linguistic contexts impact not only how we express emotions, but how we understand and conceptualize them (Bisquerra Alzina et al., 2015).

The literature on emotions and FL learning and use is quite scarce. However, previous studies looking into the expression and conceptualization of emotions in several languages have shown that emotion and emotion-laden words, especially those which carry positive connotations, can be remembered and retrieved more easily, not only in the L1, but also in the L2 (e.g., Ferré et al., 2010). Furthermore, those positive emotion words which make a biggest impact in the mind are words learned earliest (e.g., Ferré et al., 2010; Sabater et al., 2023). Several factors have been found to be modulators of emotional intensity in the L2: age of acquisition, context of acquisition, and, most importantly, proficiency (cf. e.g., Dewaele, 2008; Eilola et al., 2007; Yao et al., 2023). Accordingly, emotional intensity displays a similar behaviour in L1 and high-proficiency L2 learners, lower-proficiency learners show more detachment and are less sensitive to emotions in their L2 than in the L1 (e.g., Dewaele, 2008). Specially, when the L2 is learned in formal or academic deprived of emotional context with impoverished emotional stimuli, emotional memories are rather associated to the L1 (e.g., Robinson & Altarriba, 2014).

It is, however, difficult to discern whether similar levels of emotional intensity in L1 and L2 can be traced back to the effects of emotionality or because of the effects produced by the translation of the L1 equivalents. We might think that, as in other conceptualization examples with taxonomic and other category types (e.g., animals, and countryside), learners might “translate” or transpose the lexico-semantic and conceptual items from the L1 into the L2, especially lower-level learners. This idea would be in line with Kroll and Stewart’s (1994) *Revised Hierarchical Model* of a shared conceptual store, where L2 lexical items are accessed or activated via L1.

Empirical studies by Wierzbicka (1999) and Pavlenko (2008) have shown that different languages differ in how they conceptualize and cut up emotions and the words they have at their disposal to express those emotions. Think about the word *Schadenfreude* in German, which does not exist in English or Spanish and refers to the happiness derived from somebody’s bad fortunes, or the Danish word *hygge* for coziness, or feeling good, or well-being. The languages we speak might affect how we conceptualize emotions. Work with bilinguals and language learners has shown that L2 emotion words take more processing time (cf. Foolen, 2012), show weaker skin conductance and physiological reaction (Harris et al., 2003), and are less intense than in the L1 (Caldwell-Harris, 2014), with learning context, age of acquisition, and familiarity of use, but, mainly, L2 proficiency acting as a strong modulator of these foreign language effects (Yao et al., 2023). Studies carried out with semantic fluency or categorization tasks revealed that positive emotion categories are very productive (Blanco et al., 2020), and that they are more productive in the L1 than in the L2 (Lam & Yoon, 2023). Dewaele (2008) believes that L2 users might have incomplete emotional scripts or scenarios.

## 3. Love as a Primary and Universal Emotion

Love is considered a complex emotion, which requires a certain degree of processing, and which is impacted by the social, cultural, and linguistic context, as well as by personal experiences. In general terms, it includes different spheres such as romantic, sexual, or family love (cf. Bisquerra Alzina et al., 2015). Accordingly, Dewaele (2008) carried out an interesting study, where participants had to weigh the emotional intensity of the phrase “I love you”. He found that, for most participants, “I love you” felt strongest in their respective L1s, but FL dominance and use, and context and age of acquisition also played a role in determining the emotional intensity of love. Different cultures express love in different ways (cf. Dewaele, 2008), and, hence, the conceptualization and categorization of love might change from culture to culture, from language to language, as in what defines the love scenario or whether and how often endearment forms are used.

Altarriba and colleagues (e.g., Altarriba & Bauer, 2004) found out that emotion words such as *love* function as primes for other emotion words (*happy* and *sad*), and also for emotion-laden words such as *gift*, *teddy bear*, or *candy*, but learners with several languages were slower in their retrieval and showed less intensity (also Ferré et al., 2022). Tang (2024) examined the differences in the processing of emotion-label and emotion-laden words in L1 and L2. She found that emotion-label words produced faster reactions than emotion-laden words, but the results were comparable in both languages, L1 and L2. This might translate in emotion-label words occupying preference positions in lexical retrieval over emotion-laden words. This is yet to be probed empirically. She also found that participants exhibited less complete access to L2 emotion words compared to L1.

Previous studies dealing with the organization of the emotional lexicon for the category *love* found that Spanish and English native conceptualizations differ, with English speakers opting for more metaphoric and symbolic associations and Spanish for sensory and referential (Grabois, 1999). The conceptualization of *love* often draws on metaphorical references for processing. Yang et al. (2024) follow the principles of conceptual metaphor theory and argue that *love*, as an abstract concept, is represented in the mind via or through the help of metaphors which relate the target with concrete concepts such as *heart*, *plant*, *flower*, *rose*, *red*, or *pink*. Specifically, color metaphors are very productive when it comes to conceptualizing *love*, with *red*, mainly, but also *pink*, being associated with love in quite a culturally independent way. In the same way, the lack or loss of love are associated with grey. Their empirical study is conducted with Chinese native speakers reacting in their L1, and they wonder if similar color metaphors appear with participants from other cultural and linguistic backgrounds. Our study might shed light on this matter.

In an empirical study with lexical availability data (of the semantic fluency type), Blanco (2022) could attest that love was the most productive emotional field, i.e., the emotion than elicited most responses on the part of the Spanish L1 participants. It also elicited many different words or word types showing a moderately high cohesion index, indicating that it is a relatively compact field. He found that, among the words that conform the love scenario, the most accessible were *cariño (darling/affection and care)*, *Felicidad (happiness)*, *familia (family)*, *respeto (respect)*, *alegria (joy)*, *pareja (couple)*, *amistad (friendship)*, *afecto (fondness)*, *comprensión* (*understanding*) and *confianza (trust and confidence)*.

When the semantic activation of emotion fields or categories through semantic fluency tasks is compared between L1 and L2, the results point to L1 activation being faster and richer; i.e., more responses are yielded, and they are more strongly connected (Feng & Liu, 2024) and evoke more contextually diverse and metaphorical associations (Lam & Marquardt, 2022). In particular, L2 responses tend to show a reduced emotional intensity and simpler morphological forms when naming love-related or emotional concepts. Emotional words in L1 not only produce a more fluent output but are also linked to deeper and faster affective processing (Winskel, 2013) and greater physiological responses like increased skin conductance (Cohrssen-Hernández, 2021). The emotional category of “love” is processed more deeply and automatically in L1, unless the L2 has become dominant through high immersion and use, which may then increase the emotional resonance in L2 (Degner et al., 2012).

Research with bilinguals with migration backgrounds in their L1s or in the additional language is almost non-existent. Most studies come from the German and Spanish contexts, with findings indicating that bilinguals report different emotionality in their L1s (e.g., Turkish–German, Dewaele & Pavlenko, 2002). Additionally, L2 proficiency plays a relevant role, with highly proficient learners producing comparable emotion words in L1 and L2 (Mavrou et al., 2023). Furthermore, evidence from lexical attrition studies shows that prolonged L2 immersion can lead to sparser, less interconnected L1 semantic networks in bilinguals, especially those with immigrant backgrounds (Chaouch-Orozco & Martín-Villena, 2025), supporting the view that bilinguals’ mental lexicons are dynamically shaped by social and contextual language exposure. To date, to our knowledge, there are no studies in the literature that address mainly the expression of the emotion of *love* by monolingual EFL learners and EFL heritage bilingual learners. Furthermore, we are unaware of any research that compares the performance of these two groups or systematically examines their lexical–semantic categorization and emotional scenarios, such as the *love* scenario, in the FL, English. This study, therefore, aims to fill this gap in the existing literature. We believe it is important to research the lexical access and lexico-semantic categorization of emotions such as *love* among different EFL learners groups because it might offer insights into the mechanisms of bilingualism, and cross-cultural and cross-linguistic conceptualization of emotions. This study treats the mental lexicon as a complex cognitive system structured like a network. We believe that the way this system is organized directly influences how easily and quickly a language user can search for and recall words (lexical retrieval). In this network model, each word is a node, and the links between words (called edges) represent shared features, like semantic or formal similarity. Stronger connections (heavier edges) mean easier access to the word. This organized structure is what allows for efficient communication. By mapping this organization, this study aims to gain insight into the mechanisms of word learning, storage, and retrieval. The most important metrics used for this analysis are the degree of the node, which refers to how many other words a single word is directly connected to; the path length, which is the shortest route between two words; the clustering coefficient, which measures how interconnected a word’s immediate neighbors are and how much they form a tight group; and the modularity, which quantifies how distinctly the network divides into separate communities or clusters.

The study of semantic memory consistently employs mathematical graph theory and its associated metrics to investigate the internal organization of human language, aiming to shed light on how vocabulary is structured and retrieved from the mind (Kenett et al., 2011; Borodkin et al., 2016; De Deyne & Storms, 2008).

These studies have helped identify structural limitations that impact word learning and acquisition. A consistent finding is that the lexicon exhibits a small-world structure, comprising a dense, highly interconnected core and numerous “lexical islands”—words with sparse connections, many of which are isolated from the main network component. This configuration is considered analogous to the structure seen in other complex social and behavioral systems. From a psycholinguistic perspective, this means that highly connected words (high degree) tend to link to other high-degree words, forming tight clusters, while words with fewer links often neighbor similarly isolated terms. This unique structural density ensures that word accessibility is swift, resilient, and accurate, as the high clustering coefficient and degree provide multiple pathways for retrieval.

With the above considerations in mind, the present study pursues the following objective: to explore the conceptualization of the emotion *Love* in Spanish as L1 in monolingual learners, and Spanish as L1 in bilingual learners and English as FL or an additional language. Accordingly, we pose the following research questions:

1. How do participants conceptualize *Love* in Spanish L1 and English FL? What are the differences and similarities between L1 and FL conceptualization?
2. Are there any differences in the conceptualization of *Love* in L1 and FL between monolingual and bilingual EFL learners?

## 4. Method

This is a cross-sectional study with one single moment of data collection from intact classes and high ecological validity.

### 4.1. Sample of Participants

Informants were attending 4th of Secondary Education, year 10, they were 15–16 years old. A general proficiency test, Oxford Placement test, was conducted to inform the learners’ proficiency level, which was set at the A2^1^ level. Only participants at this level were selected so as to conform the most homogeneous groups possible in terms of proficiency, since it is believed to be a most relevant modulator or emotion understanding and expression in the L2/FL, as we have seen in the previous sections.

Participants were divided into 2 groups of 33 participants each. Group 1 is made up of monolingual Spanish EFL learners and group 2 made up of Spanish/Arabic bilingual EFL learners. Learners in this second group belong to the so-called heritage speakers. These are children born in Spain to immigrant parents who speak Arabic at home, the language of the family, but are socialized and schooled in Spanish, the language of the community. Schooling happens from age 3 onwards in Spain, which makes these learners early bilinguals who acknowledge both Spanish and Arabic as their L1, as per the answers to a demographic questionnaire completed prior to the experiment.

### 4.2. Instruments

Participants had to complete an Oxford Placement Test (computerized pen and paper version v.2) to determine their proficiency level and a demographic questionnaire. They also had to respond in Spanish (shared language of schooling) and English FL (learned as a school subject in school context) to a semantic fluency task. They had two minutes to respond to the stimuli, *LOVE* and *AMOR/AMAR*, and write as many words came to mind in relation to the prompts. Time was controlled automatically in the application. As we saw above, *Love* is an emotion which elicits many associations, and which also requires deep levels of processing and might be influenced by cultural and personal experiences.

### 4.3. Analysis and Procedures

Data were obtained online via a self-devised application in a single session, first in English and then in Spanish in order to avoid priming from the L1. Participants responded to the OPT and the fluency task at school in the period devoted to the English class. Data were curated, misspellings corrected, repeated responses per student eliminated, responses in plural singularized, and lexical phrases hyphenated so as to be analyzed as a single item, such as *teddy-bear*, or *Saint-Valentine*. Synonyms were treated as separate words.

We establish four main comparisons: how monolingual and bilingual learners respond in Spanish L1 (comparisons 1 and 2 to answer research question 1), and how monolingual and bilingual Spanish learners respond in English EFL (comparisons 3 and 4 to answer research question 2).

We apply the principles of complex network theory and graph analysis to examine the empirical data obtained. We believe graph analysis is an adequate methodology to examine fluency data, since it can serve as a proxy to the structure and organization of the mental lexicon (cf. Feng & Liu, 2024; Hernández Muñoz et al., 2025).

Specifically, we investigated total tokens produced in response to the prompts, total types produced, or number of different words. This measure is calculated on an aggregated fashion. We also examined cohesion index to check how compact and homogeneous the responses were. Additionally, we looked into the most accessible words, those which appear most frequently and first in the response list, and the most central words or anchor words, i.e., those which have the highest numbers of associations. Because we were interested in examining the nature of the organization and structure of the mental lexicon for Love and Amor, we also calculated graph metrics. Specifically, we obtained *average degree*, which represents the average number of connections (edges) per node in a graph; the *average path length*, which is the average number of steps along the shortest paths for all possible pairs of nodes in a graph; *the clustering coefficient*, which measures the degree to which nodes in a graph tend to cluster together; *sigma*, which is a measure of “small-world” characteristics in a network with values of sigma greater than 1, suggesting that the network exhibits small-world properties, meaning it has high clustering and relatively short average path lengths; and, finally, *modularity*, which is used to assess the strength of division of a network into modules or communities, that is, how well a network can be divided into densely connected groups with fewer connections between groups (cf. Hernández Muñoz et al., 2023). We used *LexPro 0.2.4* (Hernández Muñoz et al., 2023) and *Gephi* 0.10 (Cherven, 2015) for the analyses.

## 5. Results and Discussion

In the present study, we wanted to explore the way in which learners with diverse linguistic backgrounds and experiences conceptualize the emotion of *love* in one of their first languages and in the foreign language. Research question 1 asked about the differences in conceptualization between L1 and L2/FL, and research question 2 looked into the differences between monolingual and bilingual learners. Table 1 displays the descriptive results.

*Love* is a very productive field, probably because of the high impact that positive emotion words have in the mind of the learners and probably because these words are learned early in life, and, therefore, are readily accessible (Ferré et al., 2010; Frances et al., 2020; Sabater et al., 2023). Participants were quicker when responding in the L1 than in EFL, both monolingual and bilingual participants, which answers RQ1. Bilingual learners were found to be slower than their monolingual counterparts when responding in Spanish L1, but not in EFL (RQ2). Altarriba and Canary (2004) found similar results and believed that bilinguals’ access to information in several languages slowed the processing of emotion words, especially in the native languages of the bilingual speaker. The low proficiency of the learners in the L2/FL might account for (a) the fewer responses in EFL and (b) the lack of differences between monolinguals and bilinguals in the additional language.

Additionally, inferential statistics were conducted with the total number of responses per participant in order to check for significant differences among the groups. The results show, for comparison 1 (see Section 4), how Spanish monolingual learners respond to *Love* vs. *Amor*. Because both variables (responses to *Love* and responses to *Amor*) followed a normal distribution, we conducted a *t*-test, which revealed highly significant differences in favor of responses in Spanish L1 (t = −8.866, *p* < 0.001 (*p* = 3.95 × 10^−10^)). This might suggest that monolingual participants allocate a considerably higher emotional intensity to the conceptual scenario in their L1 than in the FL. Comparison 2 considered the responses by Spanish–Arabic bilingual learners. The results of non-parametric means comparisons (Wilcoxon W, because the samples do not meet a normality assumption) revealed differences in the production of responses (W = 101.0, *p* = 0.001). This likely points to bilingual participants also responding with more intensity to the emotion in one of their L1s (language of schooling, not home language) than the FL. As stated before, low proficiency in EFL might account for these differences between L1 and L2/FL and supports the argument of proficiency as a modularity factor in weighing emotionality in the FL (cf. e.g., Sabater et al., 2023).

The other two comparisons checked the similarities and differences among the two groups for the L1 and FL prompts (RQ2). Comparison 3 included responses to *Love* and the results show a lack of significant differences (Mann–Whitney U, U = 506, *p* = 0.97; the samples did not meet the normality assumption nor variance homogeneity). This result might point to monolingual and bilingual participants responding similarly in terms of emotional intensity to the emotion in the FL. Finally, comparison 4 had participants being compared when responding in Spanish L1. The results indicate a lack of statistically significant differences (U = 367, *p* = 0.08), but the descriptive results appear to suggest that monolingual participants respond slightly more intensely to the emotion in Spanish (mean 12.54 vs. 10.55) than bilingual participants.

The results of the numerical responses show that monolingual participants distinguish very notably between the conceptual scenario in L1 and FL, suggesting a higher intensity and emotional processing in their L1. This result is consistent with previous studies on emotional linguistics (e.g., Dewaele, 2008; Robinson & Altarriba, 2014). Bilingual participants show more similar levels of responses in one of the L1s and the FL (5 points of differences for the Spanish-only learners vs. 2.5 points of difference for the Spanish–Arabic speakers). The fact that Spanish is the language of school and society and not the language spoken at home might be made accountable for these results showing a slight emotional detachment in comparison with monolinguals. Both groups of participants respond in similar terms to the emotion in English, clearly revealing the impact of the schooling process in their acquisition and perception of the FL.

The fact that bilingual learners produce more responses for *Amor* than for *Love* presumably indicates that the school context may be a good source of input to develop rich associations, even if Spanish is not the home language.

In order to establish qualitative comparisons, we checked the most accessible words (50) for each participant cohort for *Love* and *Amor*. The results appear in Table 2.

As we can observe, for the 50 most accessible words, there is an overlap of 27 out of 50 (54% for *Love* between monolingual and bilingual participants), and 32 out of 50 (64%) for *Amor*. Moreover, we can observe that the equivalent words appear in *Love* and *Amor* (68% for Spanish only and 64% for Spanish–Arabic); this could be due to two main reasons. First, it could be that learners translate the words that define their emotional scenario from the L1 into the L2/FL (e.g., Chaouch-Orozco & Martín-Villena, 2025). Second, it could be simply that learners share a conceptual scenario for both linguistic systems, as suggested by Kroll and Stewart (1994) in their Revised Hierarchical Model, and that they access the L2 category via L1 translation equivalents. This is very likely since *love* is a universal emotion (Blanco, 2022).

Accordingly, some forms of endearment such as *cariño* do not appear in the English FL data, probably because there is no straightforward translation equivalent. In this sense, our observation concurs with Altarriba (2003) who found that the Spanish concept *cariño* [*liking?*, *dear*, *darling*, and *honey*] could not be translated directly into English, had no one-to-one mapping, and represented a feeling between liking and affection. Harris et al. (2006), in a study with heritage bilinguals, probed that endearment forms, such as *honey*, *sweetheart*, or *cariño*, *darling*, show the biggest L1 vs. L2/FL difference in emotional recall. Our results appear to support this claim.

Further examples of the absence of one-to-one mappings or difficulty in translation also appear in *confianza* [*confidence* and *trust*], and, also, *caricia* [*caress*] and *el enamorado* [*the person in love*], *matrimonio* [*marriage*, and *couple*], and *bombón* [*bonbon*, and *chocolate*], which do not appear in the English data, we believe, because learners do not know the translation equivalents, since they are not straightforward or one-to-one. Further support of the translation process appears with the terms *chico* [*boy*] and *chica* [*girl*] or *película* [*film*] which appear exclusively in the Spanish–Arabic data both in Spanish and in English. We believe these instances might tentatively lend support to the idea that learners, both monolingual and bilingual are resorting to L1 translation equivalents, whenever possible, to construct the L2/FL emotional category *Love*.

When the total compatibility values are considered, we observe that, for *Love*, the Spanish-only group produced a total of 96 types or different words of which 52 were exclusive and 44 shared with the Spanish–Arabic sample. The latter, in turn, produced 66 exclusive types out of the 110 total types produced. Accordingly, there is a compatibility overlap of 27.16%. The Spanish-only group included 66 singletons, that is, words produced only by one single participant; the Spanish–Arabic group elicited 69 singletons. For *Amor*, the Spanish-only group produced a total of 138 types or different words of which 82 were exclusive and 56 shared with the Spanish–Arabic sample. The latter, in turn, produced 73 exclusive types out of the 129 total types produced. Accordingly, there is a compatibility overlap of 26.54%. The Spanish-only group included 83 singletons, that is, words produced only by one single participant; the Spanish-Arabic group elicited 72 singletons. As we can see, the figures for the overlap are high. The high numbers of the singletons could likely be accounted for by the relatively small size sample.

In order to explore the qualitative nature of the different scenarios, we perform a graph analysis and obtained the following results for the main metrics and for each language and participant group:

The metric results indicate (see Table 3) that all the networks have a small-world structure; that is, they are structured in an organized and ordered way, with many connections among the words, and also strong connections, which might allow for an easy and quick navigation. The values for the Spanish L1 data are better, both for monolingual and bilingual learners, which may plausibly indicate that their *love* lexicosemantic network is more strongly and better organized in the L1. There seem to be no differences between monolingual and bilingual learners. However, two metrics in the bilingual data in English merit further observation, namely, a lower clustering coefficient and a higher modularity. The combination of these two values leads us to believe that the network of the bilinguals in the L2/FL shows slight signs of fragmentation; that is, there are communities or word clusters, which are very strongly and tightly connected, which appear systematically together in the data, but they are more loosely connected to other word clusters. This idea is supported by the lowest cohesion index of this group.

However, in order to provide more robust information, we decided to carry out inferential statistics for the degree and modularity. While the descriptive analysis of the network structures revealed minor differences, particularly regarding the average degree and modularity, the results from the inferential statistical testing did not support a conclusion of group differentiation. Specifically, neither the degree metric analysis nor the non-parametric permutation test (bootstrapping) showed a statistically significant difference between Group Spanish-only and Group Spanish–Arabic in terms of overall network connectivity. The degree comparisons revealed no significant values either for *Amor* (U = 7116, W = 13,221, *p* = .698), nor for *Love* (U = 4620, W = 9180, *p* = .130). The bootstrapping procedure yielded an observed difference of 3.9697, which falls within the null distribution, resulting in a two-tailed *p*-value of 0.5010 for Amor and −0.1818 and *p*-value (two-tailed) of 0.8490. Consequently, the null hypothesis of no difference between the groups’ network structures cannot be rejected.

At a more qualitative level, we also identified the communities and the anchor words or most central words in each community so as to compare the lexical organization and most relevant clusters for each group.

For *Love*, the Spanish-only group produced nine communities, which might point to a highly fragmented network. The Spanish–Arabic group also produced nine communities, showing the highly similar performance of both groups in the conceptualization of *Love* in EFL. The following table, Table 4, includes these communities and, in bold, the anchor word of each.

As we can see from the table above, participants in both groups share some communities and anchor words (in bold in the table); they include mainly emotion-laden words, or words that help them define and describe the *love* script, words that evoke *love* such as *friend*, and family relations, but also romantic *love*, with love metaphors such as *heart*, *butterfly*, *ring*, *chocolate*, or *flower*. The most outstanding difference is the appearance of religious love in the bilingual data, such as *God* and *paradise*, but not in the monolingual. Cultural aspects and a different understanding of the *Love* scenario among the participants from the two learner cohorts, and its implications and components, together with different experiences and contexts of language use might account for these differences.

In the case of *Amor*, in the participants L1, we could identify nine communities for the Spanish-only group and also nine for the Spanish–Arabic group (see Table 5).

In the Spanish L1 data, we can observe some poignant differences, with the Spanish–Arabic learners displaying several communities or clusters for family terms and terms related to wedding and marriage. They also include more negative terms in their description of the *Love* scenario and terms referring to physical appearance which are absent from their peers’ data. The Spanish-only participants have a more romantic and abstract perception of *Love*. They also include a community with verbs related to the emotion *Love* and another one with adjectives and another with nouns. This probably indicated a higher linguistic awareness on their part, with Spanish–Arabic learners having mixed communities as regards grammatical class.

**Figure 1:**
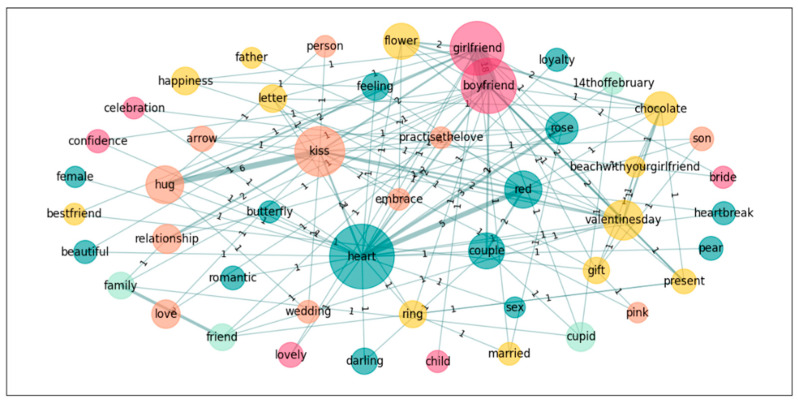
Graph for *Love*; Spanish only.^4^

**Figure 2:**
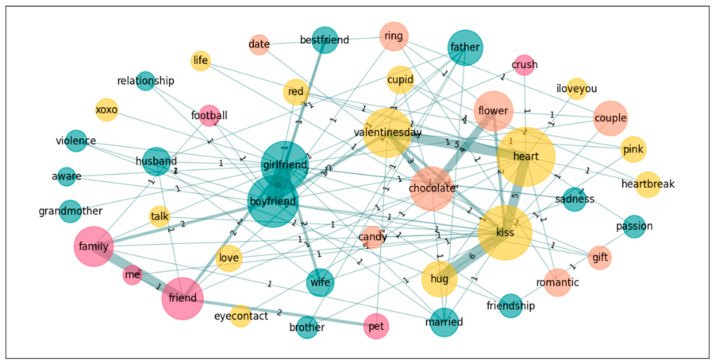
Graph for *Love*: Spanish–Arabic.

**Figure 3:**
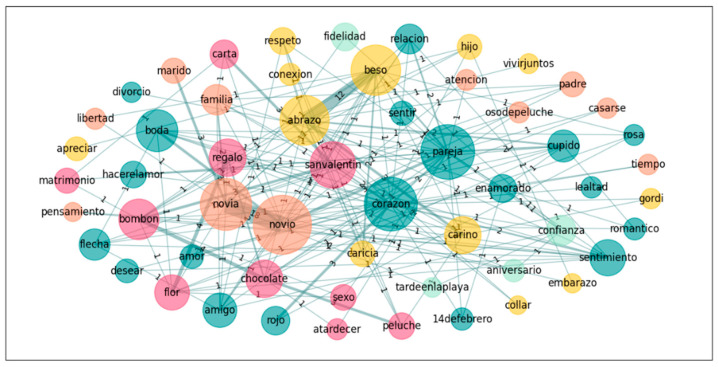
Graph for *Amor*: Spanish-only.

**Figure 4:**
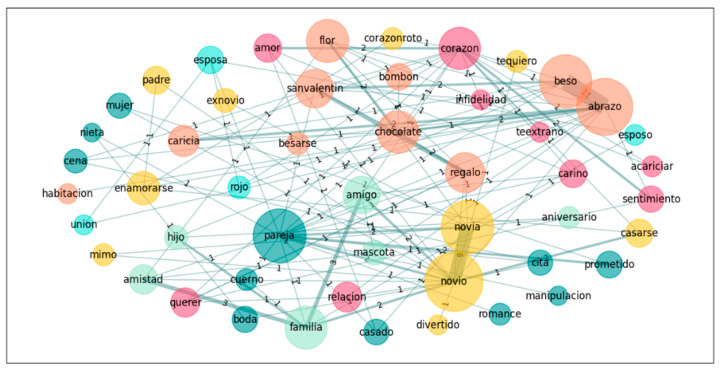
Graph for *Amor*: Spanish–Arabic.

In a further step towards exploring the nature of the groups’ conceptualization of the emotion *Love*, we also performed a graph analysis on the five most accessible words for both groups in order to see the links these words establish in the different groups. Accordingly, we calculated the subgraphs of the following words: *heart*, *boyfriend*, *girlfriend*, *kiss*, and *Valentine’s day* for *Love*; and *novio*, *novia*, *pareja*, *beso*, *corazon*^2^, and *abrazo*^3^ for *Amor* (see Table 6 and Figure 1, Figure 2, Figure 3 and Figure 4).

**Figure 5:**
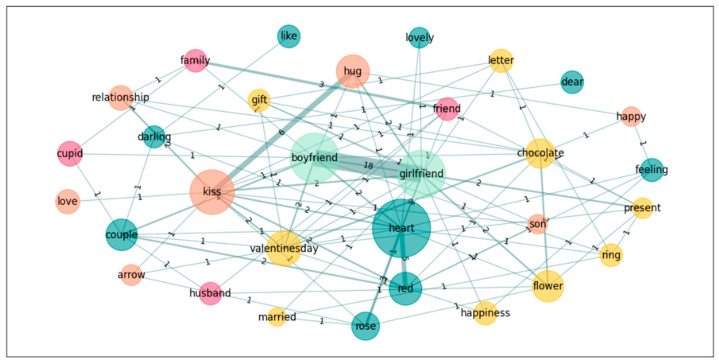
Graph for Spanish-only core responses to *Love*.

**Figure 6:**
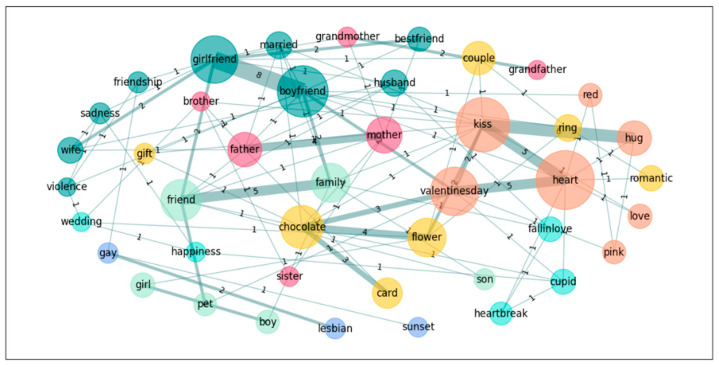
Graph for Spanish–Arabic core responses to *Love*.

**Figure 7:**
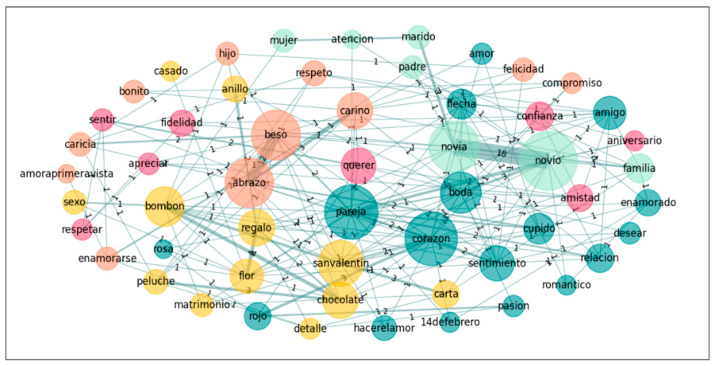
Graph for Spanish-only core responses to *Amor*.

**Figure 8:**
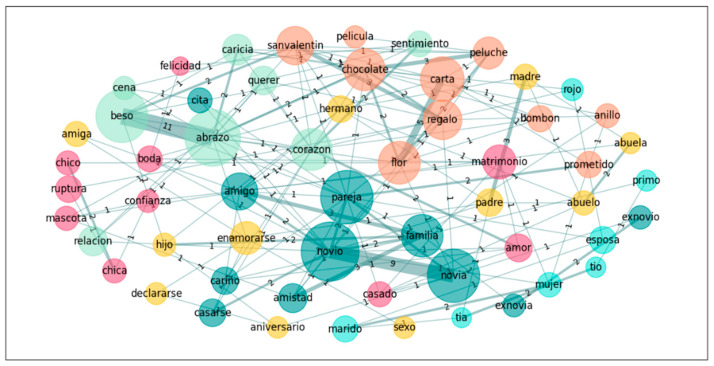
Graph for Spanish–Arabic core responses to *Amor*.

Finally, we pruned the data in order to eliminate all singletons and recalculated the graphs. This procedure mirrors those used in previous studies (e.g., Borodkin et al., 2016; Tomé Cornejo et al., 2025), where words produced only once were eliminated prior to graph analyses. The following table, Table 7, offers the results and we include the graph illustrations as well (see also Figure 5, Figure 6, Figure 7 and Figure 8).

The data from the most central words and from the core clusters abound in the ideas that (1) the L1 network is more strongly connected and better organized to facilitate navigation, and (2) that the bilinguals’ network displays a very tightly connected core and loose connections to other clusters. The connections in the monolingual data seem to be more homogeneously distributed; this might indicate a more efficient navigation. These metrics notwithstanding, we acknowledge the mainly qualitative and exploratory nature of our analysis.

It is interesting to note that our results mirror previous findings in that *love* is a very productive field, both in L1 and L2/FL, although slightly less in the L2/FL. Additionally, it is a field which elicits a high number of different responses or word types, which might point to learners’ creativity and recourse to their different experiences to conceptualize *love*. Our results are also in line with Blanco (2022) in terms of the most accessible and readily available words, which include (in L1 and L2/FL): *family*, *respect*, *dear* and *darling*, *joy*, *happiness*, or *confidence* and *trust*. We believe these are learned early in life and describe the main *love* scenario for our participants. Interestingly enough, the results for the most accessible words also concur partially with the results from the ANEW project on emotional stimuli norms (ANEW database (Bradley & Lang, 1999) adapted to Spanish L1 by Redondo et al. (2007)).

The research consistently finds that bilinguals and monolinguals generate different sets of words in fluency tasks due to the differences in exposure and conceptual mappings. For example, Dubé and Thordardottir (2024) showed that, even when the total word counts are similar, the types and uniqueness of words differ significantly by language exposure and background, reinforcing the value of capturing group-specific vocabulary. In this sense, we need to insist on the idea that the cultural salience and frequency effects of the lexical items might be brandished as alternative explanations of possible differences beyond monolingual/ bilingual status prior to EFL learning.

Our results mirror those of previous studies (e.g., Sabater et al., 2023) in that emotion-laden words, such as *dolphin*, *gift*, and *candy*, are more frequent in the learners’ data than emotion-label words, such as *sad*, *happy*, and *hurt*, since the former belong to the same conceptual space within *love*, and the latter represent distinct conceptual spaces. Since these are learned earlier in life (Sabater et al., 2023), they are more readily accessible when retrieved in the semantic fluency task. This is congruent with our results and with the idea that emotion-laden words are a true and valid resource when representing and conceptualizing emotions—*love*, in our case.

In our data, we believe learners who are of low proficiency are attaching new labels, i.e., L1 translation equivalents in the L2/FL, to old concepts; thus, we see how learners describe the same or very similar scenarios in their L1 and L2/FL, e.g.,: *gift*, *chocolate*, *teddy bear*, *family*, *friend*, *boyfriend*, and *girlfriend*. This might be in line with the RHM where learners access their L2/FL concepts via L1. This might be compatible with our data in that responses were fewer in the L2/FL than in the L1, probably because of a lack of usage and of emotional ground. L2/FL affective words might have less direct or automatic access or be connected via L1 words, and this might explain why one-to-one mappings of translation equivalents are so frequent in our data (cf. Yao et al., 2023). Other researchers (see Pavlenko (2008)) have also argued that aspects of the L1 concept or of the emotion scenario might be transferred to the L2/FL conceptual scenario, especially when the L2 is learned in formal contexts only. Our results also seem to concur with this observation.

Additionally, learners appear to resort to figurative language or metaphors to conceptualize *love*, like in *bridge*, *education*, *chemistry*, *heart*, *plant*, *flower*, *rose*, *red*, or *pink*. Figurative language has an important conceptualizing function of emotions which can be found both in L1 and L2/FL in both monolingual and bilingual EFL learners (Foolen, 2012). The fact that there is relational complexity within the field of *love*—cause, experience, target, and effect (cf. Foolen, 2012)—and that many emotion-laden words related to *love* are abstract, metaphors, and metonymies, for instance, *heart*, emerge as a useful resource to express and conceptualize *love*, as our data suggest. The use of metaphor might not be conscious, but it clearly helps users describe their emotional experiences in more accurate ways. Metaphor appears both in L1 and L2/FL responses, probably supporting the L1 translation theory, even of metaphorical domains, brandished above. A special note is due concerning color metaphors related to the abstract concept *love*. As in previous studies, our data reveal that learners resort to color metaphors to express and represent *love*, such as *red* or *pink* (Yang et al., 2024), and this can be argued to be true for both Spanish L1 and English L2/FL, and for monolingual and bilingual learners, which adds to the idea that the color metaphorical representation of *love* is quite culturally independent as pointed out previously by Yang et al. (2024). They argue that extended cultural practices related to, for instance, red lipstick to enhance sensuality, wearing red on Saint Valentine’s Day, and gifting red roses on love anniversaries help explain this association. We believe our results lend support to these ideas.

## 6. Conclusions

The present study sought to answer two research questions: first, how participants differed in their conceptualization of *Amor* (L1) and *Love* (L2/FL); and, second, how monolingual and bilingual learners differ in the conceptualization of *Love* and *Amor* in the L2 and the shared L1. In answer to the first question, we could arguably see that the lexicosemantic network of *love* is richer in the L1 than in the L2/FL. The semantic representation of *love* in the L2 tends to be highly mediated by the L1 culture and language. The concept of *love*, we believe, is deeply rooted in early linguistic experiences, with *love* words and *love*-related words learned early in life, and cultural experiences. The conceptualization of *Love* appears to mirror that of *Amor*, not only via L1 translation equivalents, but also by reproducing metaphorical representations. The answer to the second question is twofold. Both learner cohorts display similar conceptualizations of *Love* in EFL, although they differ very slightly in how they react to *Amor* in the shared L1, Spanish, probably because they lack home experience with Spanish. However, the results of the graph analysis revealed a lack of significant differences between the groups as concerns their vocabulary network structure. We have, nonetheless, provided some qualitative analysis that, in merely descriptive terms, serves to shed light on the differences and similarities of how both specific populations conceptualize the emotion of *Love* in their L1 and EFL.

To our knowledge, this is the first study that looks into the conceptualization of *Love* in L1 and EFL of monolingual learners and bilingual learners with a migration background and especially using the principles and techniques of graph analysis, which allow for deep insights into the structure of the emotion lexicon of learners. Although mostly descriptive, we believe that this study can add to the existing knowledge in the field of semantic categorization and lexical access, specifically within the realm of the expression of emotions in the additional language. Furthermore, the exploration of the mental lexicon of learners with a migration origin in the school language and in EFL is a rare enterprise, and our research might serve to raise awareness of the topic and stimulate interest in the issue.

The main limitation of the study pertains to the lack of data in the bilinguals’ other language, Arabic. Further research could try to cover this gap and look into the conceptualization of other emotions such as *happiness*, *anger*, *surprise*, or *fear*.

## Figures and Tables

**Table 1:** Descriptive results.

Variable	Love		Amor	
	Spanish-Only	Spanish–Arabic	Spanish-Only	Spanish–Arabic
Total tokens	257	263	414	348
Types	96	110	138	129
Mean tokens	7.78	7.97	12.54	10.55
Cohesion index	0.08	0.07	0.09	0.08

**Table 2:** Most accessible words.

Love		Amor	
Spanish Only	Spanish–Arabic	Spanish Only	Spanish–Arabic
Heart	Heart	Novio	Abrazo
Boyfriend	Kiss	Pareja	Novio
Girlfriend	Boyfriend	Corazón	Beso
Kiss	Valentinesday	Novia	Pareja
valentinesday	Girlfriend	Beso	Novia
Hug	Chocolate	Abrazo	Carta
Red	Friend	Sanvalentin	Chocolate
Couple	Family	Boda	Flor
Flower	Flower	Bombon	Corazón
Chocolate	Mother	Chocolate	Sanvalentin
Rose	Hug	Regalo	Regalo
relationship	Father	Cariño	Familia
Cupid	Couple	Querer	Peluche
Love	Card	Sentimiento	Caricia
happiness	Ring	Flor	Amigo
Letter	Married	Amigo	Enamorarse
feeling	Husband	Cupido	Relacion
Ring	Wife	Flecha	Matrimonio
Like	Romantic	Familia	Amistad
Dear	Cupid	Confianza	Querer
family	Fallinlove	Relacion	Cariño
Friend	Girl	Rojo	Amor
husband	Love	Carta	Ruptura
Gift	Bestfriend	Amistad	Hermano
darling	Boy	Enamorado	Prometido
Arrow	Red	Fidelidad	Bombon
Lovely	Pink	Anillo	Padre
present	Heartbreak	Respeto	Cita
You	Pet	Hacerelamor	Boda
romantic	Friendship	Peluche	Esposa
Crush	Son	Sexo	Mascota
Hurt	Sadness	Caricia	Chica
Pear	Gift	Marido	Anillo
Card	Lesbian	Felicidad	Chico
happy	Book	Matrimonio	Casarse
Son	Gay	Enamorarse	Madre
confidence	Happiness	Padre	Cena
friendship	Passion	Hijo	Sentimiento
Wife	Horny	Mujer	Abuelo
Enjoy	Xoxo	Bonito	Confianza
beautiful	Fantastic	Sentir	Película
heartbreak	Film	Apreciar	Rojo
myboo	Sunset	Desear	Romántico
loyalty	Grandmother	Gustar	Besarse
wedding	Sister	Pasión	Teamo
Help	Wedding	Amor	No
Married	Bombastic	Respetar	Casado
Baby	Person	Detalle	Declararse
Feel	Teddy	Compromiso	Aniversario
Child	Videogame	Encantar	Romance

**Table 3:** Graph metrics for the complete network.

		Average Degree	Average Path Length	Clustering Coefficient	Sigma	Modularity
Love	Spanish-Only	3.73	3.67	0.15	3.89	0.46
	Spanish–Arabic	3.38	4.26	0.09	3.9	0.52
Amor	Spanish-Only	4.36	3.78	0.12	4.55	0.47
	Spanish–Arabic	3.95	3.72	0.10	4.43	0.53

**Table 4:** Communities for *Love* (anchor words in bold).

Spanish Only	Spanish–Arabic
**Kiss**, hug, wedding, person, pink, fall in love	**Kiss**, heart, Valentine’s day, hug, cupid, red, heartbreak, passion, crush, pink, cry, emotion, eye contact, I miss you
**Couple**, husband, cupid, 14th February, child, father, bridge, lovesong, wife	**Chocolate**, **flower**, gift, candy, love, talk, card, teddy, music, comfortable, letter, honest
**Valentine’s Day**, **chocolate**, flower, gift, letter, bestfriend, embrace, bouquet, candy, detail, house, dollar, beach with friends	**Father**, mother, videogame, confident, holiday, sensitive, star
**Arrow**, lovely, red, heart, need, angel, bow	**Boyfriend**, girlfriend, bestfriend, violence, relationship, husband, wife, relationship, sadness, fall in love, exgirlfriend
**Heart**, red, rose, feeling, son, happy, heartbreak, romantic, anniversary, daughter, loyalty, female, male, help, care, sex, hurt	**Friend**, family, married, son, pet, boy, happy, poem, balloon, football, world, engaged, happy life, God, paradise
**Girlfriend**, boyfriend, ring, married, confidence, celebration, bride	**Couple**, brother, wedding, sister, happiness, grandmother, gay, lesbian, sunset, grandfather, uncle, transsexual, sunrise, sad, horny
**Friend,** family, relationship, present, respect, friendship, bestie, pet, time, crush, sweetheart	**Ring**, romantic, date, marriage, fantastic, bombastic, amazing, good, sex
**Darling,** happiness, love, like, dear enjoy, feel, incredible, true love, spectacular, baby, sweetie, a lot, so much	**Film**, book, person, romance
**Butterfly**, attention, self-love, date, film	**Food**, train, time, quality, respect, peace, money, clothes

anchor words in bold.

**Table 5:** Communities for *Amor*.

Spanish Only	Spanish–Arabic
**Novio**, novia, amigo, familia, padres, marido, mujer, casarse, divorcio, libertad, embarazo, casa	**Pareja,** novio, novia, familia, amigo, hijo, amistad, prometido, mascota, casarse, cita, rubia, manipulación, violencia, rubia, ojos azules
**Beso**, cariño, abrazo, bombón, chocolate, hijo, caricia, peluche, felicidad, respeto, sexo, atardecer, afecto, amor eterno, amor incondicional, ramo de flores	**Abrazo**, chocolate, san Valentin, regalo, flor, carta, beso, peluche, bombón, caricia, cupido, romance
**Confianza**, enamorado, aniversario, fidelidad, romántico, compromiso, luna de miel, bonito, risa, diversión, enamorada, cuernos, manipulación, seguridad, cumplido, ayuda	**Confianza**, chico, chica, ruptura, poema, aprendizaje, distancia, gay, lesbiana, fiesta, celos, toxico
**Querer**, amistad, sentir, apreciar, respetar, gustar, lealtad, conexión, disfrutar, confiar, regalar, extrañar, encantar, gustar, hermosura, educación, química, aprecio	**Matrimonio**, boda, mujer, cena, casado, marido, nieta, charla, poema, enamoramiento, pedida de mano, confesión
**Flor**, regalo, detalle, enamorarse, anillo, casado, suegro, suegra, vivir juntos, prometido	**Enamorarse**, esposa, aniversario, declararse, sexo, corazón roto, futbol, pasión, religión, tarta de boda
**Corazón**, pareja, sentimiento, boda, San Valentin, cupido, flecha, relación, carta, rojo, matrimonio, rosa, pasión	**Corazón**, querer, sentimiento, amor, cariño, relación, rojo, felicidad, infidelidad, acariciar, romántico, emoción, traición, libre,
**Amar**, prometida, besar, abrazar	**Hija**, relación sexual, prueba de amor
**Amor a primera vista,** cita, flechazo, cena, celebración, tolerancia, caballerosidad	**Película**, libro, habitación, cine, música, chuches
**Atención**, dedicación, demostración, plan, viaje	**Hermano**, padre, abuelo, madre, primo, abuela, tia, tio, hermana, prima, tatarabuela

anchor words in bold.

**Table 6:** Graph metrics for the most accessible words.

		Average Degree	Average Path Length	Clustering Coefficient	Sigma	Modularity
Love	Spanish-Only	4.58	2.30	0.36	2.64	0.36
	Spanish–Arabic	4.19	2.43	0.31	1.91	0.40
Amor	Spanish-Only	5.75	2.34	0.30	2.34	0.37
	Spanish–Arabic	4.48	2.47	0.23	2.44	0.40

**Table 7:** Graph metrics for the core network.

		Average Degree	Average Path Length	Clustering Coefficient	Sigma	Modularity
Love	Spanish-Only	5.48	2.19	0.32	1.66	0.31
	Spanish–Arabic	4.42	2.78	0.19	1.9	0.45
Amor	Spanish-Only	6.47	2.34	0.26	2	0.39
	Spanish–Arabic	5.19	2.70	0.18	2.35	0.46

## Data Availability

The raw data supporting the conclusions of this article will be made available by the authors on request.

## Notes

1 Participants’ results in the OPT were as follows, and revealed no significant differences among the two learners cohorts: Proficiency level scores in EFL. For Spanish-only learners had a mean of 25.56 (S.D. 6.2), Spannish-Arabic learners obtained a mean score of 24.48 (S.D. 4.8), Wilcoxon signed-rank test values were: (W = 1507, *p* = .737).

2 It appears in 10th position of accessibility for the Spanish–Arabic group.

3 It appears in 6th position of accessibility for the Spanish-only group.

4 In the graph, Figure 1, Figure 2, Figure 3, Figure 4, Figure 5, Figure 6, Figure 7 and Figure 8, different colors indicate the different communities, the size of the node indicates degree or number of connections, and the numbers attached to the edges indicate number of repetitions of the association or bigram.
